# Supplementary material for: Inhibitory Effect of Eslicarbazepine Acetate and S-Licarbazepine on Nav1.5 Channels
Source: Front Pharmacol. 2020 Oct 2;11:555047. doi: 10.3389/fphar.2020.555047 (PMC7567166; doi:10.3389/fphar.2020.555047)
Supplement: Supplementary file 2 [file DataSheet_2.docx]

**Supplementary Figure Legends**

**Supplementary Figure 1.** Effect of 0.45% DMSO on VGSC current-voltage relationship and gating in MDA-MB-231 cells. (A) Current-voltage (I-V) plots of Na^+^ currents in MDA-MB-231 cells in physiological saline solution (PSS; black circles) and in PSS with 0.45% DMSO (0.45% DMSO; green squares). Currents were elicited using 10 mV depolarising steps from -80 to +30 mV for 30 ms, from a holding potential of -120 mV. Results are mean ± SEM (n = 13-17). (B) Activation and steady-state inactivation in physiological saline solution (PSS; black circles) and in PSS with 0.45% DMSO (0.45% DMSO; green squares). For activation, normalised conductance (G/G_max_) was calculated from the current data and plotted as a function of voltage. For steady-state inactivation, normalised current (I/I_max_), elicited by 50 ms test pulses at -10 mV following 250 ms conditioning voltage pulses between -120 mV and +30 mV, applied from a holding potential of -120 mV, was plotted as a function of the prepulse voltage. Results are mean ± SEM (n = 10-13). Activation and inactivation curves are fitted with Boltzmann functions.

**Supplementary Figure 2.** Effect of 100 μM eslicarbazepine acetate on Na_v_1.5 currents. (A) Representative Na^+^ currents in an MDA-MB-231 cell elicited by a depolarisation from -120 mV to -10 mV in physiological saline solution (PSS; black), eslicarbazepine acetate (ESL; 100 μM; red) and after washout (grey). Dotted vertical lines define the time period magnified in (B). (B) Representative persistent Na^+^ currents in an MDA-MB-231 cell elicited by a depolarisation from -120 mV to -10 mV. (C) Representative Na^+^ currents in an MDA-MB-231 cell elicited by a depolarisation from -80 mV to -10 mV. (D) Normalised Na^+^ currents in MDA-MB-231 cells elicited by a depolarisation from -120 mV to -10 mV. (E) Normalised Na^+^ currents in MDA-MB-231 cells elicited by a depolarisation from -80 mV to -10 mV. (F) Representative Na^+^ currents in a HEK-Na_v_1.5 cell elicited by a depolarisation from -120 mV to -10 mV in PSS (black), ESL (100 μM; red) and after washout (grey). Dotted vertical lines define the time period magnified in (G). (G) Representative persistent Na^+^ currents in a HEK-Na_v_1.5 cell elicited by a depolarisation from -120 mV to -10 mV. (H) Representative Na^+^ currents in a HEK-Na_v_1.5 cell elicited by a depolarisation from -80 mV to -10 mV. (I) Normalised Na^+^ currents in HEK-Na_v_1.5 cells elicited by a depolarisation from -120 mV to -10 mV. (J) Normalised Na^+^ currents in HEK-Na_v_1.5 cells elicited by a depolarisation from -80 mV to -10 mV. Results are mean + SEM. *P ≤ 0.05; **P ≤ 0.01; one-way ANOVA with Tukey tests (n = 7-8). NS, not significant.

**Supplementary Figure 3.** Effect of 100 μM S-licarbazepine on Na_v_1.5 currents. (A) Representative Na^+^ currents in an MDA-MB-231 cell elicited by a depolarisation from -120 mV to -10 mV in physiological saline solution (PSS; black), S-licarbazepine (S-Lic; 100 μM; red) and after washout (grey). Dotted vertical lines define the time period magnified in (B). (B) Representative persistent Na^+^ currents in an MDA-MB-231 cell elicited by a depolarisation from -120 mV to -10 mV. (C) Representative Na^+^ currents in an MDA-MB-231 cell elicited by a depolarisation from -80 mV to -10 mV. (D) Normalised Na^+^ currents in MDA-MB-231 cells elicited by a depolarisation from -120 mV to -10 mV. (E) Normalised Na^+^ currents in MDA-MB-231 cells elicited by a depolarisation from -80 mV to -10 mV. (F) Representative Na^+^ currents in a HEK-Na_v_1.5 cell elicited by a depolarisation from -120 mV to -10 mV in PSS (black), S-Lic (100 μM; red) and after washout (grey). Dotted vertical lines define the time period magnified in (G). (G) Representative persistent Na^+^ currents in a HEK-Na_v_1.5 cell elicited by a depolarisation from -120 mV to -10 mV. (H) Representative Na^+^ currents in a HEK-Na_v_1.5 cell elicited by a depolarisation from -80 mV to -10 mV. (I) Normalised Na^+^ currents in HEK-Na_v_1.5 cells elicited by a depolarisation from -120 mV to -10 mV. (J) Normalised Na^+^ currents in HEK-Na_v_1.5 cells elicited by a depolarisation from -80 mV to -10 mV. Results are mean + SEM. *P ≤ 0.05; ***P ≤ 0.001; one-way ANOVA with Tukey tests (n = 7-8). NS, not significant.
