# Supplementary figures and images for: Inhibitory Effect of Eslicarbazepine Acetate and S-Licarbazepine on Nav1.5 Channels
Source: Front Pharmacol. 2020 Oct 2;11:555047. doi: 10.3389/fphar.2020.555047 (PMC7567166; doi:10.3389/fphar.2020.555047)

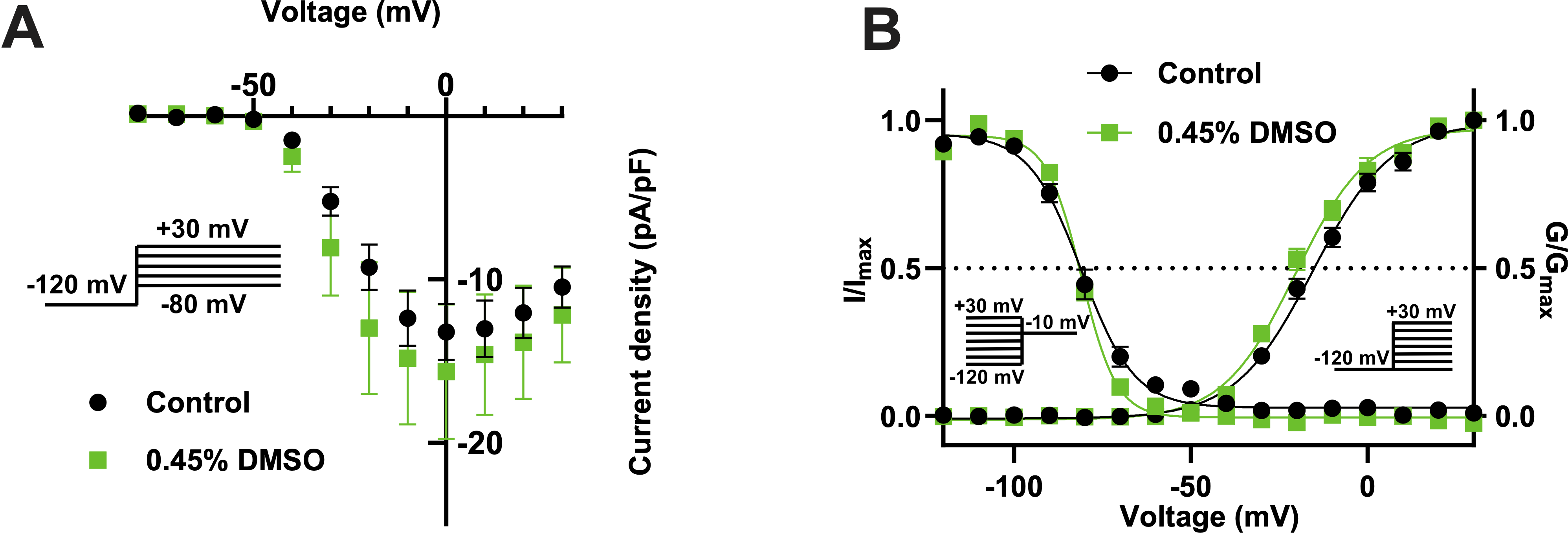

Supplement: Supplementary file 3 [file Image_1.tiff]

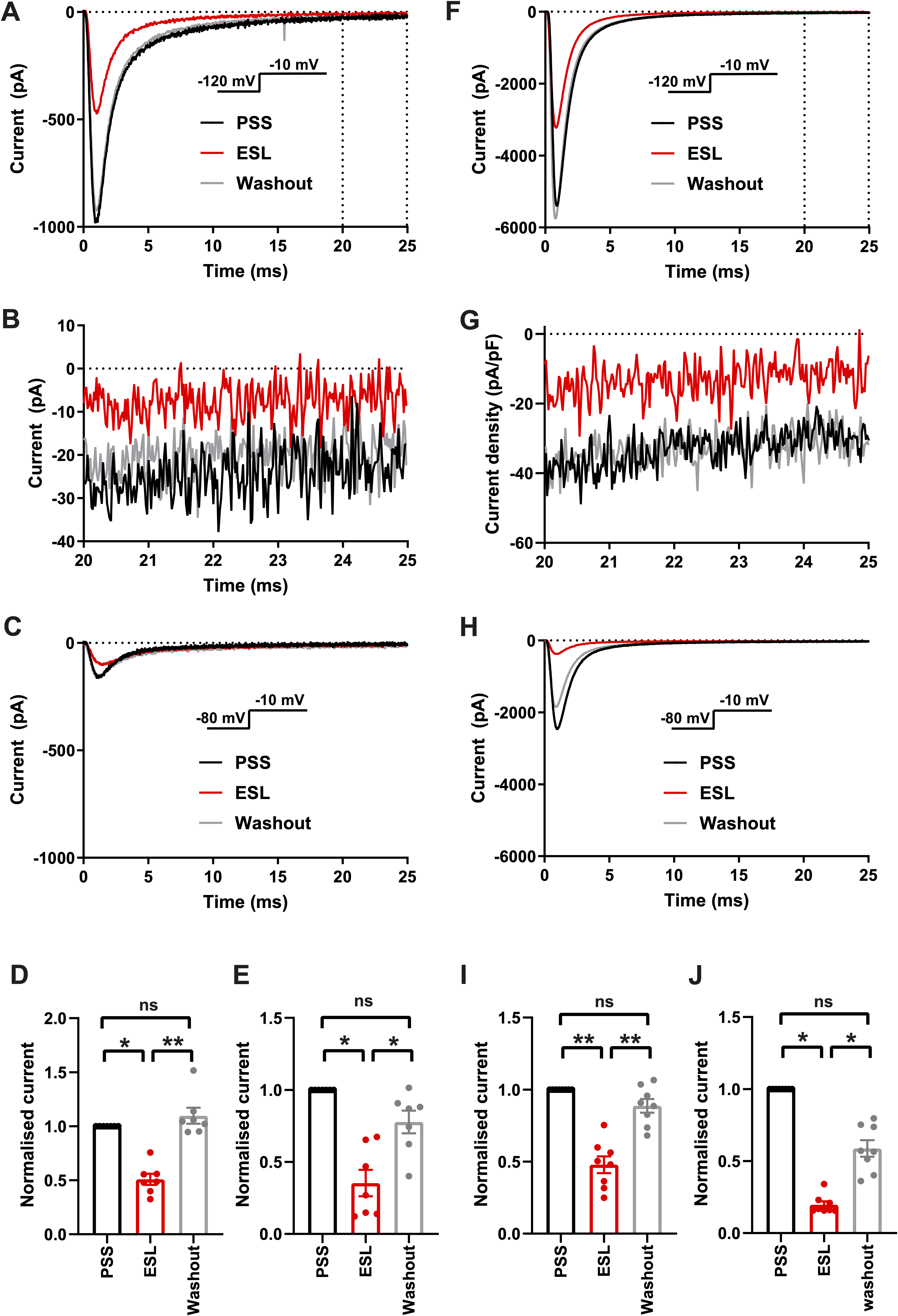

Supplement: Supplementary file 4 [file Image_2.tiff]

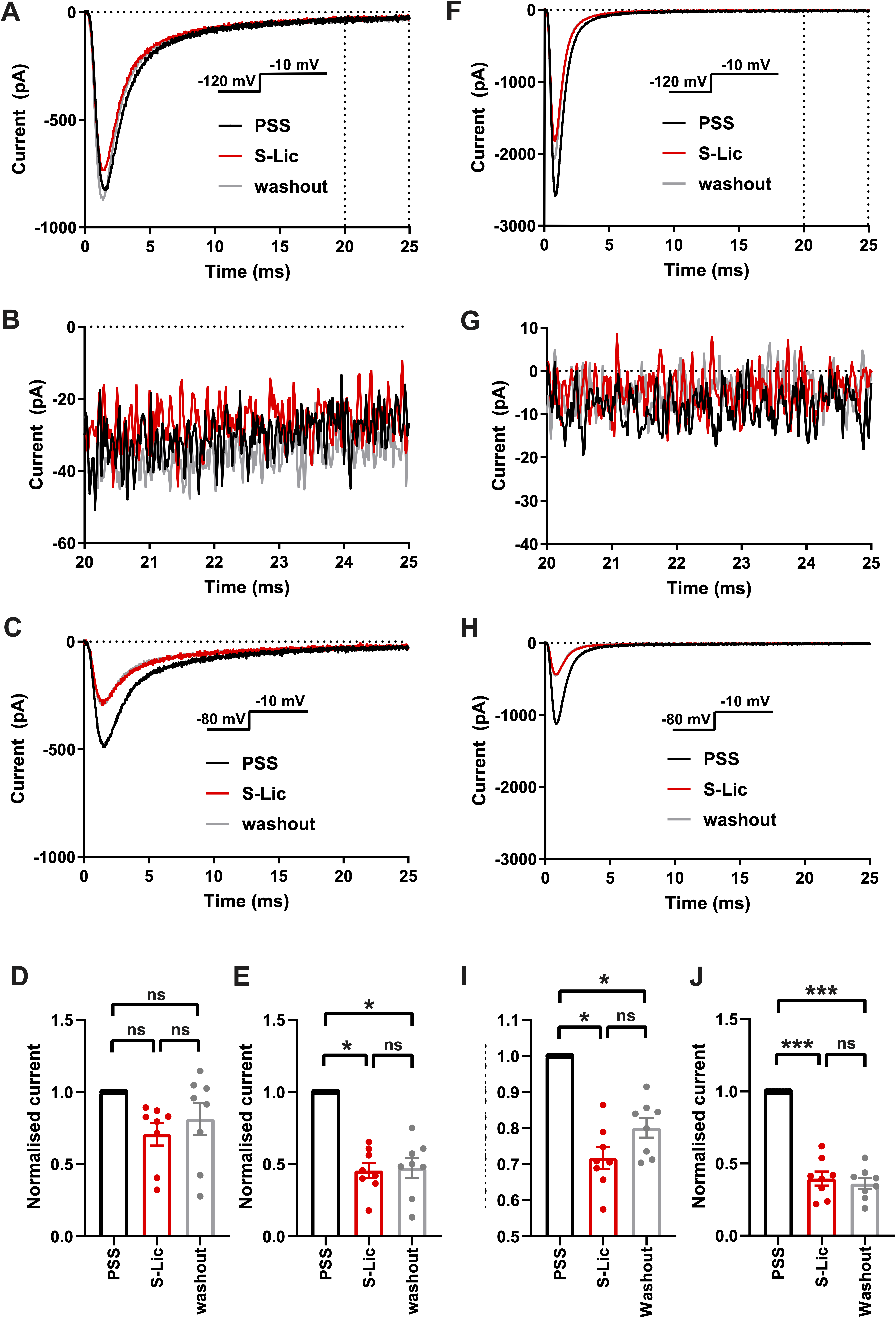

Supplement: Supplementary file 5 [file Image_3.tiff]
